# Using technology to support diabetes care in hospital: Guidelines from the Joint British Diabetes Societies for Inpatient Care (JBDS‐IP) group and Diabetes Technology Network (DTN) UK

**DOI:** 10.1111/dme.15452

**Published:** 2024-10-21

**Authors:** Parizad Avari, Pratik Choudhary, Alistair Lumb, Shivani Misra, Gerry Rayman, Daniel Flanagan, Ketan Dhatariya, the Joint British Diabetes Societies for Inpatient Care (JBDS‐IP) group

**Affiliations:** ^1^ Department of Diabetes and Endocrinology Imperial College Healthcare NHS Trust London UK; ^2^ Department of Metabolism, Digestion and Reproduction Imperial College London London UK; ^3^ Diabetes Research Centre University of Leicester Leicester UK; ^4^ Oxford Centre for Diabetes, Endocrinology and Metabolism Churchill Hospital Oxford UK; ^5^ Ipswich Diabetes Centre East Suffolk and North East Essex NHS Foundation Trust Ipswich UK; ^6^ Department of Endocrinology University Hospital Plymouth Plymouth UK; ^7^ Elsie Bertram Diabetes Centre Norfolk and Norwich University Hospitals NHS Foundation Trust Norwich UK; ^8^ Norwich Medical School University of East Anglia Norwich UK

**Keywords:** continuous subcutaneous insulin infusion, diabetes, hospital, hybrid close loop, inpatient, insulin pump, wearable technology

## Abstract

This article summarises the Joint British Diabetes Societies for Inpatient Care (JBDS‐IP) Group guidelines on the use of technology to support diabetes care in hospital. The guideline incorporates two main areas: (i) use of wearable technology devices to improve diabetes management in hospital (including continuous glucose monitoring and insulin pump therapy) and (ii) information technology. Although it is reasonable to extrapolate from the evidence available, that devices developed to enhance diabetes care outside hospital will show similar benefits, there are challenges posed within the inpatient setting in hospital. This guidance provides a pragmatic approach to supporting self‐management in individuals using wearable technology admitted to hospital. Furthermore, it also aims to provide a best practice guide for using information technology to monitor diabetes care and communicate between health professionals.

**What's new?**

- There is significant variation in the use of all aspects of technology for diabetes management in hospital, with lack of national guidance in the UK.
- This guideline provides a pragmatic approach to supporting self‐management with use of wearable diabetes technology in hospital and provides a best practice guide for information technology in hospital.
- Supporting education and awareness of diabetes technology across multiple specialisms for clinicians and healthcare professionals will be paramount for successful implementation within the hospital setting.

## 1 INTRODUCTION

Over the past couple of decades, there has been a dramatic rise in the use of technology to improve the lives of people with diabetes. These devices have mainly been developed for use outside of hospital, demonstrating improved glycaemia,^1^, ^2^, ^3^ increased satisfaction, reduced fear of hypoglycaemia,^4^, ^5^ improved quality of life^5^ and reduced admissions with diabetes emergencies.^6^, ^7^ However, this evidence base almost entirely excludes individuals who are acutely unwell or require hospital‐based treatment. Given over a quarter of all people admitted to hospital have diabetes, there is a need for clinical guidelines for when people using them are admitted to hospital.^8^

In parallel to developments in wearable technology, significant development has been made in the way clinical information can be shared and analysed by the person with diabetes and health professionals. These include electronic patient records (EPRs), electronic prescribing, and automated alerts for identifying people with diabetes or dysglycaemia who are in hospital. This has profound implications for diabetes hospital teams requiring a different way of working to provide optimal care. Improving glycaemia, whilst minimising the rate of hypoglycaemia, is of major importance in the hospital setting, as both hyperglycaemia and hypoglycaemia have been shown to be associated with worse clinical outcomes and mortality.^9^, ^10^

The Joint British Diabetes Societies for Inpatient Care (JBDS‐IP) group and Diabetes Technology Network UK (DTN‐UK) Technology Working Group conducted a national survey on the current state of diabetes technology use and knowledge across inpatient diabetes teams in the United Kingdom. Responses were received from 42 organisations representing 104 hospitals across the UK. Overall significant variation was found between organisations in the use of technology to support safe, effective inpatient diabetes care (Table 1).^11^ The JBDS‐IP have, therefore, developed guidance to support people using diabetes technology in hospitals with a view to improving safety and outcomes of inpatient diabetes care.

**TABLE 1:** Survey responses from hospitals across UK.

Presence of specialist diabetes support with knowledge of technology	
Weekdays–normal working hours	100%
Weekdays–out of hours	64.3%
Weekends	53.7%
Specific policy for inpatient use of CGM	16.7%
Specific policy for insulin pump or hybrid closed‐loop use in hospital	64.3%
System to flag people with diabetes on admission	40.5%
System which can flag those at increased risk of harm	28.6%
Electronic system for referral to the diabetes specialist team	73.1%
Presence of networked blood glucose meters	85.7%
Data from networked meters used for audit/quality improvement	58.3%
Presence of networked blood ketone meters	76.3%

Abbreviation: CGM, continuous glucose monitoring.

This summary of the JBDS‐IP guidance is a pragmatic approach to support technology use that can be used with the current evidence base.^12^, ^13^, ^14^, ^15^ The guidance covers use of wearable technology (CGM, insulin pumps and hybrid closed loops), as well as optimising information sharing across the health care system, and making full use of data from networked glucose and ketone meters. Parts of this overall guidance have been previously published as part of the JBDS‐IP scoping reviews.^12^, ^13^, ^14^, ^15^ A summary of the key recommendations is provided in Table 2.

**TABLE 2:** Key recommendations for use of technology in hospital.

Summary recommendations: Hospitals should have a written policy for the use of wearable technologies within the inpatient setting. These include CGM, insulin pumps, and hybrid closed‐loop systems Hospitals should have a policy for POCT of networked glucose and ketone meters Hospitals should routinely use the data from networked glucose and ketone meters to enhance inpatient diabetes care and should be regularly reviewed for audit/quality improvement People with diabetes should be involved with the development of relevant hospital policies Hospital management should ensure electronic prescribing and medicines administration are available, as well as enabling the ability to integrate diabetes technology with electronic health records The hospital should ensure relevant staff training in the use of diabetes technology Policies on use of technology in hospital should undergo annual review and audit
Wearable diabetes technology: For any person with diabetes admitted to hospital, particularly T1D and insulin treated T2D, check whether they use any wearable technology Determine whether the device is a continuous glucose monitoring system (real‐time or intermittently scanned CGM) or an insulin delivery system (i.e. insulin pump) If admitted unconscious, check for wearable diabetes technology (usually worn on the arm or abdomen, but may sometimes be on thighs/ buttocks) Ensure the device (CGM/ insulin pump) is not inserted into area of generalised oedema or cellulitis
Continuous glucose monitoring: If the person with diabetes can self‐manage and are capable of using their technology device, they should be encouraged to do so as they do out of hospital Currently, CGM can be used to augment capillary glucose testing in hospital but cannot replace it. If a sensor is being used in hospital, at least two CBG tests should be performed. Otherwise, routine point‐of‐care CBG testing should be done at the previously recommended frequency (i.e. before meals and at bedtime for those on a basal‐bolus insulin regimen) For in‐hospital glycaemia, aim should be for no episodes of hypoglycaemia and to minimise hyperglycaemia Glucose between 4.0–6.0 mmol/L is indicative of looming hypoglycaemia so consider intervening, particularly if there is a downward CGM arrow Alarms should be used to trigger a capillary glucose reading and consideration of intervention by ward nursing staff If the person is due for a procedure or operation where it is agreed or planned to continue using their device, ensure it is on a different area of the body (contralateral side) so that it is not affected Avoid placing CGM sensors on the abdomen in the prone individual, as increased pressure whilst lying on it may reduce sensor accuracy Any CGM devices removed, should be labelled, stored in a safe place and documented Diabetes inpatient teams are encouraged to maintain a supply of sensors available to support people in hospital who rely on these for self‐management (although individuals are recommended to bring their own CGM supplies)
Insulin pump/continuous subcutaneous insulin infusion: An insulin pump should be discontinued if there is any impairment to consciousness, or if the person with diabetes is acutely unwell and/or confused If there is disruption of insulin delivery via subcutaneous insulin pump (for example, removal of pump or blocked cannula), ensure an alternative source of insulin is started immediately (intravenous or subcutaneous injections) Any removed insulin pump devices, should be labelled, stored in a safe place and documented All people using insulin pumps should be discussed with a member of diabetes specialist team
Hybrid closed loop: Closed‐loop algorithms should be ‘disengaged’ and switched to ‘manual’ control in hospital After discontinuation of auto‐mode within the hybrid closed loop, the system may be used individually (as CGM only or insulin pump only) if criteria are met For inpatients meeting the criteria to continue insulin pump and CGM therapy, continuing in closed‐loop mode may be considered but only under specific guidance from the diabetes team
Electronic prescribing and medicines administration: All hospitals should aim towards implementing electronic health records and EPMA Specific training should be provided for EPMA systems, insulin prescriptions, as well as care bundles, such as DKA or HHS Point‐of‐care glucose and ketone results should be integrated in to EPR systems
Point‐of‐care testing: Capillary blood glucose is the standard for glucose monitoring in hospital Capillary blood ketone should be favoured over urine testing, due to their potential for increased accuracy and connectivity (urine ketones may lag behind blood ketones) Provision and quality assurance (internal quality control in the ward environment and external quality assurance) of all POCT devices should be overseen by the Clinical Biochemistry Point‐of‐Care Committee Staff training should be standard for the use of POCT devices including the identification of erroneous results and internal quality control POCT devices should be implemented with end‐to‐end connectivity across device, middleware, laboratory information management system (LIMS) and electronic health records (EHR) Inpatient diabetes teams should be supported to use data from networked POCT devices to prospectively identify harms and people at‐risk, as well as retrospectively undertake quality improvement and benchmarking Aim to include prompts for out‐of‐range blood glucose and ketones within EHR and make them available to healthcare professionals caring for people with diabetes
Information Technology to support inpatient diabetes care: Inpatient teams should have access to a list of all people with diabetes in hospital Inpatient teams should have the ability to maintain an electronic record of people with diabetes currently under their care Data within this record needs to be auditable There should be a system of electronic referral to the diabetes team There should be an electronic system to monitor foot checks with referral to the inpatient foot multidisciplinary team as required Inpatient teams should review lists of out of range glucose and ketone readings in order to aid prioritisation of people for review (for examples, recurrent hypoglycaemia or elevated ketones) EPMA reduces diabetes related prescribing errors; prescribing information should be available to the diabetes team remotely Routine observations such as blood glucose and ketones should be monitored electronically with action prompts for the ward teams if out of range Using IT to perform regular audits of performance should be part of routine care delivery Contribution to national electronic audits of inpatient care should be part of routine care delivery
Self‐management protocols If an individual normally uses an insulin pump or glucose sensor and is confident and capable of continuing with this in hospital, then hospital systems should enable this to happen The diabetes team need to be available to closely support this process 7 days per week Point‐of‐care capillary glucose measurements performed by the hospital team may be required in addition to the individual's own glucose recordings

Abbreviations: CBG, capillary blood glucose; CGM, continuous glucose monitoring; EPMA, electronic prescribing and medicines administration; HER, electronic health records; LIMS, laboratory information management system; POCT, point of care testing; T1D, type 1 diabetes.

## 2 WEARABLE DIABETES TECHNOLOGY

**FIGURE 1:**
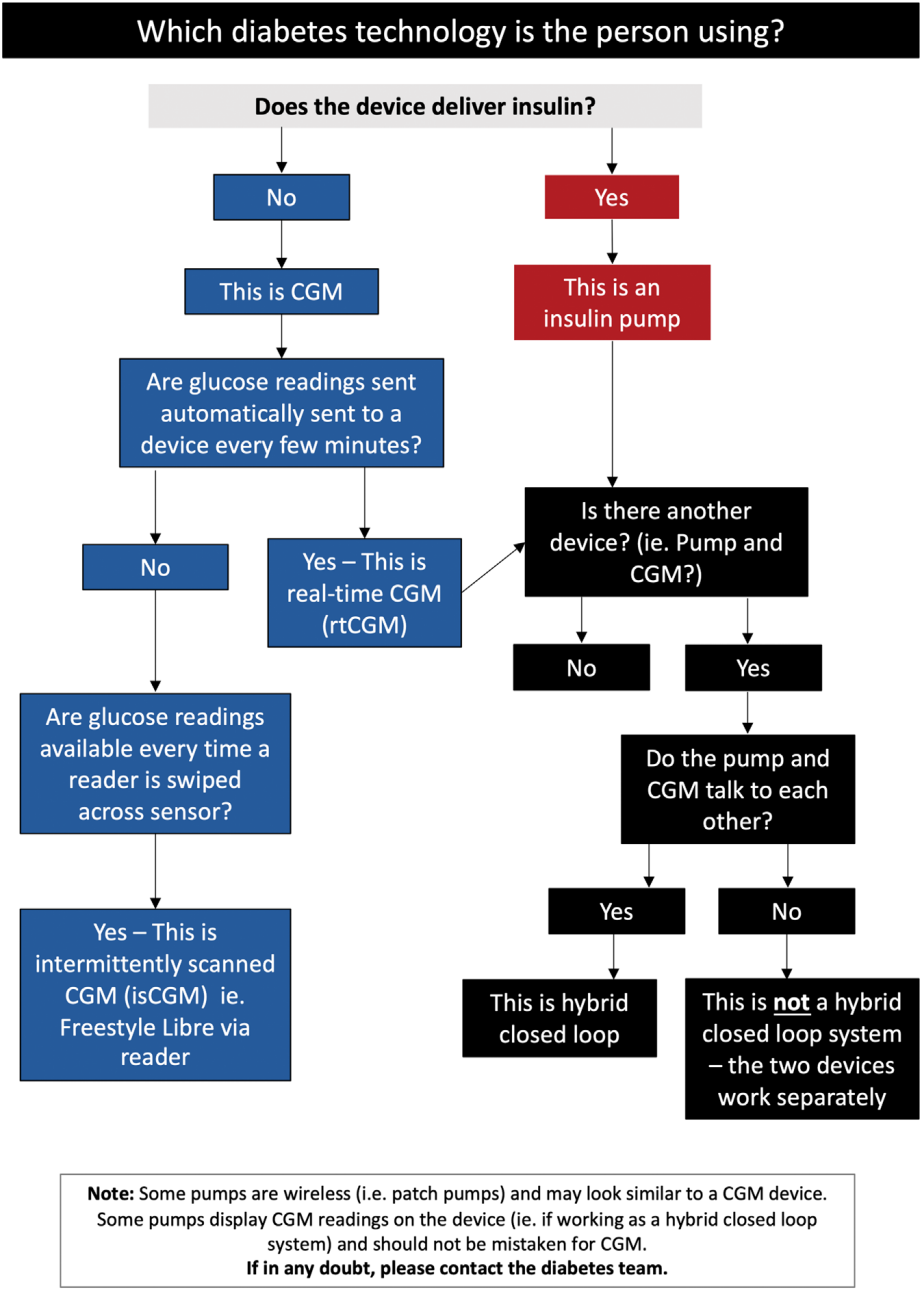
Identifying which diabetes technology is being used.

Healthcare professionals within the hospital are required to be able distinguish between the two main types of technology: CGM and insulin pumps (Figure 1). Upon hospital admission of a person with diabetes, it is important to check for any wearable diabetes technology; if unconscious, sites (i.e. arm, abdomen, or occasionally on thighs or buttocks) should be checked.

### 2.1 Continuous glucose monitoring

Continuous glucose monitoring (CGM) in the hospital setting has the potential to mitigate increased length of stay and adverse outcomes^16^ associated with raised glucose levels and hypoglycaemia by helping people in hospital and health care professionals.

Similar to the outpatient setting, the use of alarms to alert the individual as well as the healthcare team to hypo‐ or hyperglycaemia can reduce these extremes of glucose that are associated with increased length of stay and adverse outcomes. We know that much of hypo and hyperglycaemia is asymptomatic and unrecognised, therefore, improved monitoring can help identify more episodes and leading to earlier intervention and improved glycaemia.

However, there are several challenges to the routine use of CGM in hospital.^17^ Our survey highlighted the lack of familiarity of ward staff with the technology and concerns about accuracy whilst people are acutely unwell or undergoing major surgical procedures (this is due to an absence of evidence). In view of CGM devices measuring interstitial glucose, this could potentially be affected by temperature, hydration status, rapid changes in blood glucose and tissue perfusion. There are also challenges in diabetes specialist teams being able to monitor CGM remotely in hospital, as CGM is not integrated directly within current EHRs. There is also a reliance on the person with diabetes having the capacity to self‐monitor and provide accurate readings while unwell. There is also lack of high‐quality data supporting the use of CGM in the hospital setting and many of our recommendations are based on expert opinion and small observation studies.

The current gold standard for glucose monitoring and diagnostic purposes remains laboratory blood glucose. Thus, for monitoring purposes, capillary blood glucose (CBG) via point‐of‐care testing (POCT) remains the mainstay of glucose monitoring in hospital. Further studies are required on feasibility and accuracy of CGM use in hospital before they become widely used as replacements for capillary glucose testing.

#### 2.1.1 Glycaemic targets in hospital

The Advanced Technologies & Treatments for Diabetes (ATTD) consensus guidance recommends a target percentage time in range (3.9–10 mmol/L) in the outpatient setting of 70%,^18^ but this is reduced to 50% for those at higher risk of hypoglycaemia.

**FIGURE 2:**
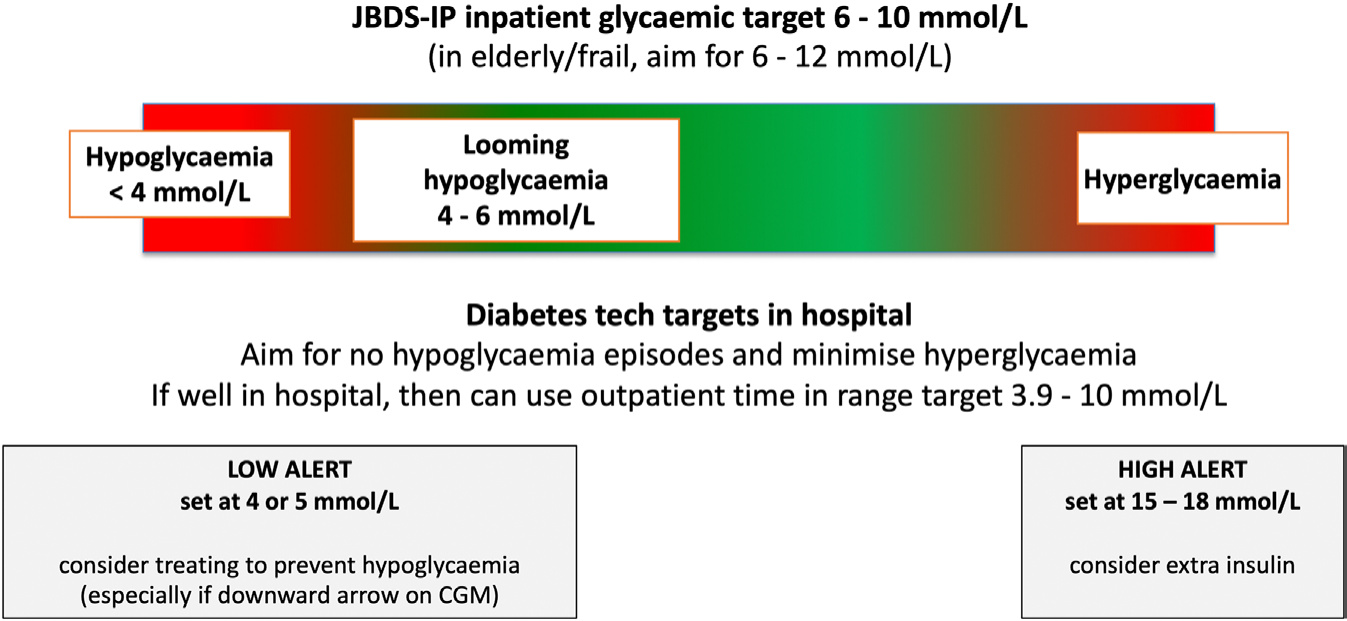
Glycaemic parameters and targets for people with diabetes in hospital.

The JBDS‐IP view is that during acute illness and inpatient stays, avoiding hypoglycaemia is the priority (aim for no hypoglycaemia in hospital), with the secondary aim of avoiding significant hyperglycaemia (Figure 2). The usual JBDS‐IP glycaemic targets in hospital are 6–10 mmol/L, which apply for the acutely unwell person or 6–12 mmol/L in the elderly/frail.

If glucose is between 4 and 6 mmol/L and CGM arrow(s) are trending down, this is indicative of looming hypoglycaemia. A small carbohydrate snack (4–8 g) can arrest the fall to hypoglycaemia. Other interventions may include adding glucose to an intravenous infusion or simply rechecking blood glucose levels sooner than planned.

#### 2.1.2 Alarms and alerts in hospital

Within the hospital setting, alarms may be challenging, particularly when healthcare professionals are unfamiliar with the technology. Currently, alarms are not integrated within electronic health records or the nursing station, and as such, the device is primarily utilised as a self‐management tool. If there is a CGM alarm, adults in hospital should be instructed to notify nursing staff immediately (either in person or via their nurse‐call button), prompting a CBG check. In order to avoid potential delays and lack of context with call buzzers, nursing staff should be aware to prioritise CGM alarms and follow local clinical guidelines for intervention based on CBG results.

If the person is able and willing to self‐manage, alarm settings may be kept as in the outpatient setting. To avoid the burden of high alarm frequency, JBDS‐IP suggest alarms to be used as safety nets to require action, rather than efficacy.

If CGM is being used to alert the medical team, the settings can be programmed as:

- HIGH ALERT: set at 15–18 mmol/L—consider extra insulin.
- LOW ALERT: set at 4–5 mmol/L—consider treating to prevent hypoglycaemia (especially if the downward arrow is shown on the CGM).

As clinical teams gain more experience, and it becomes possible to link these systems with hospital electronic health records, this can enable deliver improvements in inpatient to on out‐of‐range glucose readings. At present, the alarms should be used to trigger a CBG reading and consideration of intervention by the ward nursing staff according to clinical advice.

#### 2.1.3 Capillary glucose monitoring whilst the individual wears CGM

As CGM data are yet not integrated within electronic health records, and sensor inaccuracies may occur during acute illness, CGM can supplement but not replace CBG monitoring in hospitals. Thus, JBDS‐IP recommends capillary blood glucose should be checked at least twice daily for people using CGM in hospital, irrespective of whether the device needs calibration. It should be explained to the person with diabetes, that regular CBG monitoring is necessary for safety reasons, and for alerting staff to out of range results. Nursing staff should also be aware to perform additional CBG testing in case of any concerns of discrepancy with symptoms.

#### 2.1.4 Discrepancies between CGM and CBG readings

Potential sensor inaccuracies or discrepancies between CGM and CBG readings may be observed. JBDS‐IP define an acceptable difference based on the definition for the reference standard for integrated CGM (iCGM) devices.^19^ This is defined as %20/20 (i.e. if difference between readings is ±20% for CBG >5.6 mmol/L or within ±1.1 mmol/L (±20 mg/dL) if ≤5.6 mmol/L).

If discrepancies are observed, more frequent CBG monitoring is advised is for next few hours (depending on clinical need).

For CGM devices that can be calibrated (e.g. Dexcom G6®) consider calibration with point‐of‐care glucose using a blood glucose meter and use if accurate. If the discrepancy persists, sensor should be removed, and replaced.

Table 3 outlines situations in which people using CGM in hospital should be advised to check capillary blood glucose before treatment decisions (rather than using sensor glucose). Reasons for ensuring CBG checks are performed in hospital include that these are quality assured, and that point‐of‐care glucose results may be linked to safety systems and visible to the diabetes team.

**TABLE 3:** Situations to always check fingerstick CBG whilst using a CGM device.

Situations to always check capillary blood glucose (CBG) To confirm hypoglycaemia AND monitor recovery from hypoglycaemia If symptoms do not match sensor glucose (e.g. if symptoms of hypoglycaemia are present but the sensor glucose reading is normal) If the sensor reading seems unlikely in the circumstances If the sensor reading is unreliable or obviously erroneous (e.g. no reading, or no arrow) If required for calibration During and after exercise (e.g. after extensive physio session) When following ‘sick day rules’ or ‘rules for management of unexplained hyperglycaemia’

#### 2.1.5 Documentation of glucose readings in hospital

Currently, CGM data are not integrated with most hospital electronic health records, and therefore, healthcare professionals need to be mindful of how data are documented. Due to the influx of glucose data available from CGM devices, JBDS‐IP advocate a minimum standard of documentation including fasting, pre‐meal and bedtime readings. Instances of hypoglycaemia and hyperglycaemia, flagged by alarms, should also be recorded, along with the methodology employed for obtaining glucose values.

Diabetes teams receiving automated alerts from CBG monitoring should be mindful that until CGM systems are fully integrated with electronic health records, they will not receive alerts on out‐of‐range glucose values as with POCT glucose systems.

Historic CGM data can be viewed through appropriate sensor online platforms (Supplementary Material S1).

#### 2.1.6 The person with diabetes wearing CGM in hospital

**FIGURE 3:**
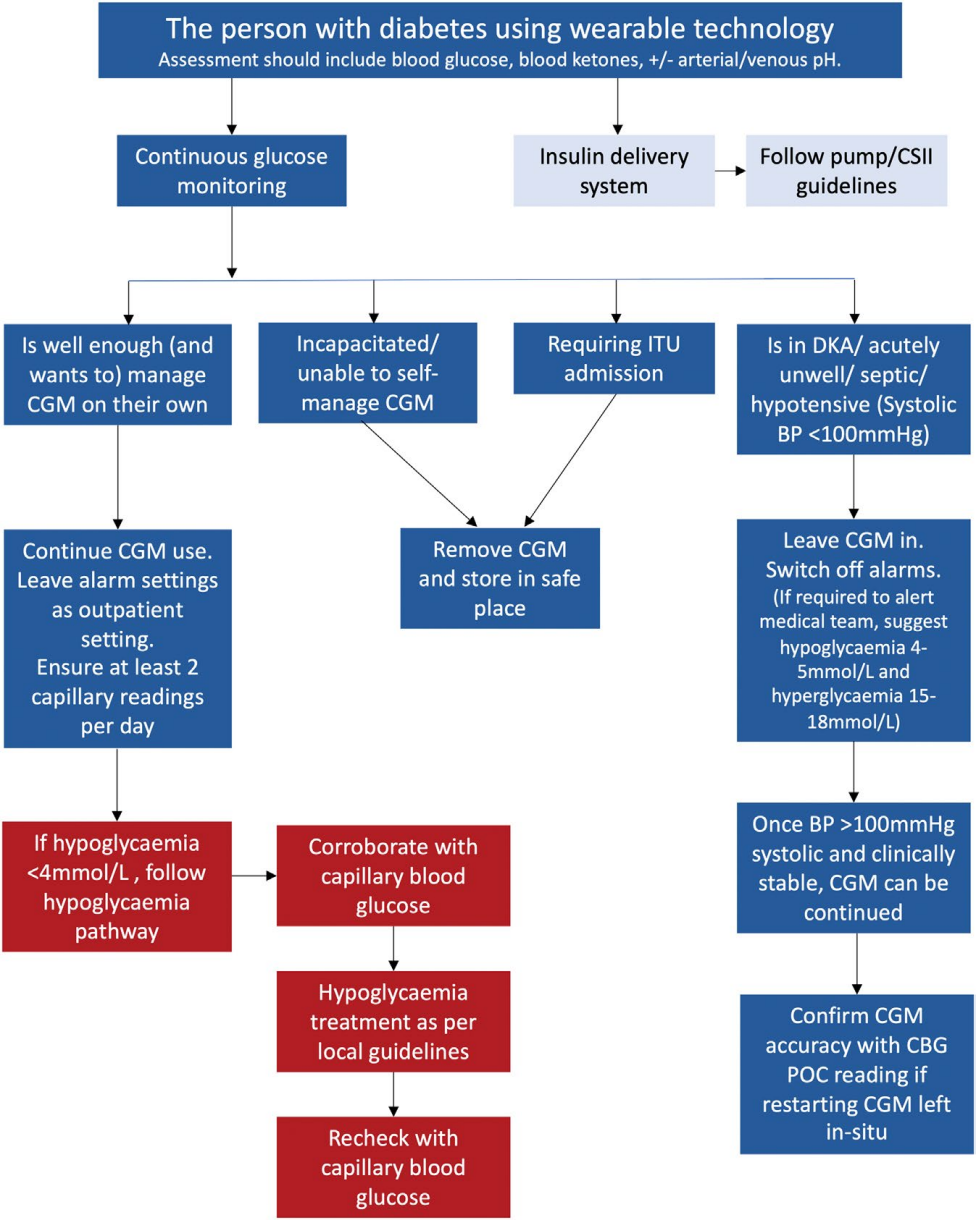
Recommendations for use of CGM in the hospital setting.

Unless incapacitated or acutely unwell (i.e. haemodynamically unstable or septic), most people using CGM in a medical or surgical ward are safe to remain on CGM if admitted to hospital.^20^ Figure 3 outlines the recommendations for use of CGM in hospital. Capillary blood glucose should be checked at least twice daily for people using CGM in hospital, irrespective of whether the device needs calibration.

**FIGURE 4:**
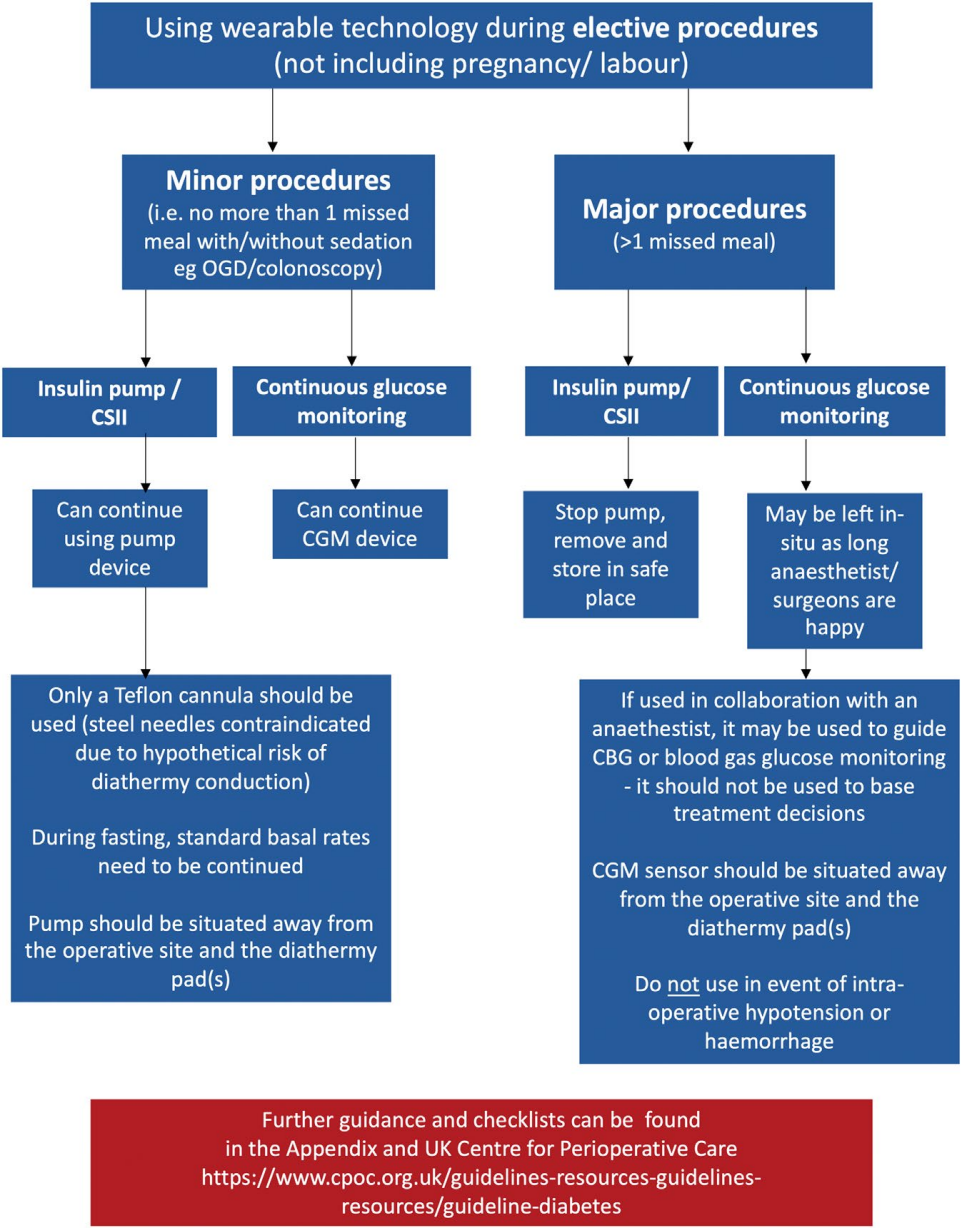
Wearable technology during elective procedures.

The CGM sensor device can be left in (unless the duration of illness is longer than the manufacturer's guidance on sensor wear). Table 4 summarises use of CGM use in special situations, with Figure 4 summarising wearable technology during elective procedures. Key points include:

- Ensure that the CGM device is not inserted into area of generalised oedema or cellulitis.
- CGM glucose readings should not be used whilst on variable rate intravenous insulin infusion (VRIII)/fixed rate intravenous insulin infusion (FRIII).
- If the patient is due for a procedure or operation, ensure the sensor is on a different area of the body (contralateral side) so that it is not affected.

**TABLE 4:** CGM and insulin pump use in special situations.

CGM	Insulin pump
In the septic, unwell individual	
Do not use CGM in those acutely unwell or haemodynamically unstable Revert to standard point‐of‐care CBG monitoring CGM devices may be left in situ Once stabilised with systolic BP > 100 mmHg and individual able to self‐manage, CGM may be continued on ward Reassess and confirm CGM accuracy before using the sensor	Do not use subcutaneous insulin pump in those acutely unwell or haemodynamically unstable Stop insulin pump, remove and store in safe place Replace with intravenous (VRIII) or multiple daily insulin injections
During hyperglycaemic emergencies	
Do not use CGM during hyperglycaemic emergencies CGM devices may be left in situ Do not use CGM readings whilst on VRIII/FRIII Capillary blood glucose monitoring should be performed to adjust insulin infusions	Do not use subcutaneous insulin pump during hyperglycaemic emergencies Insulin pump should be removed and replaced with intravenous insulin as per local protocol (FRIII/VRIII) Do not restart insulin pump until reasons for the hyperglycaemic emergency have been determined and pump equipment has been checked
In ITU	
CGM should not be used in the ITU setting	Insulin pump should not be used in the ITU setting
Radiological investigations	
MRI—All CGM devices should be removed (except the implantable Eversense® sensor) CT—Can be either removed or covered with a lead shield	MRI—Insulin pump should be temporarily suspended and removed^a^ CT—Can be either removed or covered with a lead shield FDG‐PET—No bolus insulin via pump <4 hours prior to procedure. Basal insulin can be continued through pump (discuss in advance with Radiologist) X‐rays—no need to remove the pump
During elective surgery and/or procedures	
CGM may be used to guide capillary or blood gas glucose monitoring CGM should not be used to base treatment decisions CGM sensor should be situated away from the operative site and the diathermy pad(s) Do not use in event of intra‐operative hypotension or haemorrhage Minor procedures (e.g. OGD/colonoscopy) CGM can be continued	Major surgical procedures (>1 missed meal): Stop insulin pump, remove and store in safe place Ensure alternative strategy for insulin delivery appropriate for major surgery (VRIII) Minor procedures (no more than 1 missed meal with/without sedation, for example OGD/colonoscopy) Can continue using insulin pump^b^ Only a Teflon® cannula should be used (steel needles contraindicated due to hypothetical risk of diathermy conduction) During fasting, standard basal rates may be continued Insulin pump should be situated away from the operative site and the diathermy pad(s) Ensure VRIII prescription and basal insulin is prescribed in case of pump failure Further guidance and checklists found in Figure 4 and UK Centre for Perioperative Care guidelines: https://cpoc.org.uk/guidelines‐resources‐guidelines‐resources/guideline‐diabetes
During pregnancy and labour	
Can be used safely during pregnancy and labour delivery Do not use to guide maternal VRIII Ensure sensor is moved to the arm prior to caesarean section, so does not interfere with the operative field	Can be used safely during pregnancy and labour delivery If the mother‐to‐be (or partner) does not feel confident managing the insulin pump during labour, or if blood glucose not appropriately controlled, then VRIII should be started instead Consider starting VRIII if two consecutive blood glucose levels are above the target range (7.0 as per NICE or 8.0 mmol/L as per JBDS‐IP guidance) After delivery, revert to pre‐pregnancy basal infusion rates to minimise risk of hypoglycaemia
Pacemakers	
There is an absence of data on use of pacemakers with CGM and more needs to be known with regards to safety, accuracy and compatibility	
Cardiac arrest	
CGM devices should ideally be removed for external DC cardioversion (but do NOT delay CPR) Do not use CGM glucose to guide treatment of hypoglycaemia in cardiac arrest	Insulin pump should ideally be removed for external DC cardioversion (but do NOT delay CPR)

Abbreviations: CBG, capillary blood glucose; CGM, continuous glucose monitoring; CPR, cardiopulmonary resuscitation; DKA, diabetic ketoacidosis; FRIII, fixed rate intravenous insulin infusion; ITU, intensive care unit; IV, intravenous; MRI; magnetic resonance imaging; POCT, point of care testing; SC, subcutaneous; VRIII, variable rate intravenous insulin infusion.

^a^ Pumps can be safely suspended/removed for up to an hour at a time without needing alternative insulin. A correction bolus may be needed on reconnecting the pump.

^b^ Prerequisites for safe perioperative use must be met.

For any episodes of hypoglycaemia, the person with diabetes in hospital should inform nursing staff. Point‐of‐care capillary blood glucose should be performed to corroborate the result and treated as per local guidance.

Although there are increasing data supporting use of CGM in the intensive care unit (ITU),^21^, ^22^, ^23^, ^24^ concerns include lack of accuracy in critically ill patients with impaired tissue perfusion,^25^ hypotension, hypothermia, hypoxia, vasopressor use, and potential substance interference.^26^ Currently within the UK, the major barrier for incorporating CGM systems within the critical care setting is implementation. The lack of a 24/7 diabetes team and infrastructure to support in majority of UK hospitals^11^ remains a challenge to effective implementation within critical care.

#### 2.1.7 Stopping and restarting CGM

As many non‐diabetes healthcare professionals may be unfamiliar with the technology devices, the person with diabetes is ideally best placed to remove the sensor (ideally with the phone app or receiver to be informed of sensor session ending). If this is not possible, the sensor may be removed and safely stored.

As some sensors have the transmitter attached, any devices removed, should be labelled, and stored in a safe place and documented. This should ensure that the relatively expensive transmitter is not discarded. Even though the implantable sensor (Eversense®) is not available in many parts of the world, including the UK, the surface transmitter can be removed and reapplied at a later date.

Similarly, for restarting CGM, the person with diabetes is ideally best placed to restart this as they will have received training and will be experienced in this process.

#### 2.1.8 Insulin pumps/continuous subcutaneous insulin infusion therapy

Overall, there are limited randomised studies, although an observational study indicating significantly fewer episodes of severe hyperglycaemia (>16.7 mmol/L) and hypoglycaemia (<2.2 mmol/L) in people in hospital using a pump.^27^ No difference was observed in mean glucose between those continuing continuous subcutaneous insulin infusion therapy (CSII) and those not.^27^, ^28^ However, giving individuals autonomy to self‐manage glycaemia where possible, may also reduce the risk of insulin administration errors. People often request to continue CSII use and report high satisfaction (86%) when allowed to do so.^29^

**FIGURE 5:**
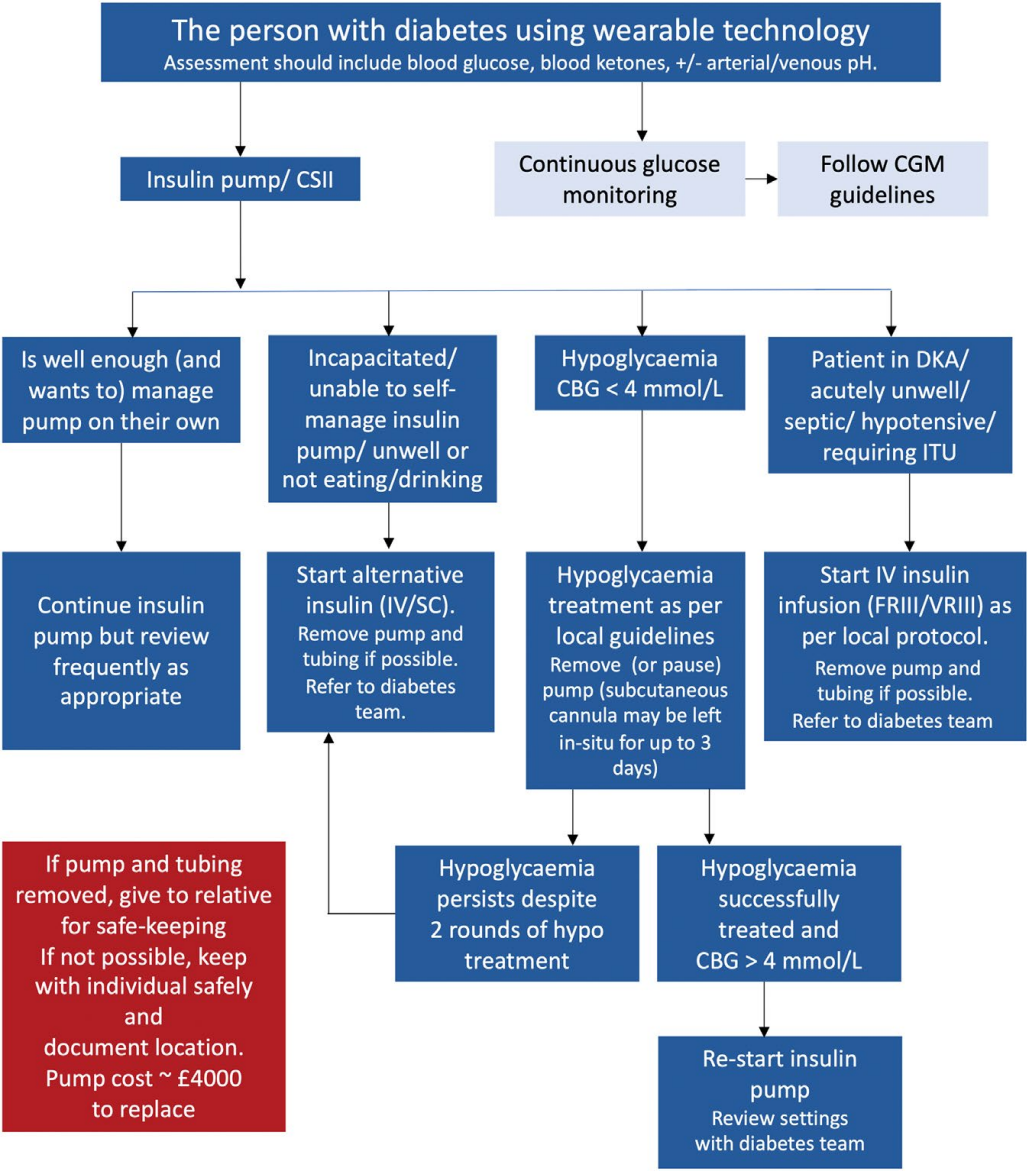
Recommendations for use of insulin pumps in hospital.

On acute admission to hospital, a considered decision, involving the person with diabetes, should be made as to whether the person can continue to safely use their pump and whether they wish to do so (Figure 5). The key criteria for insulin pump continuation include:

1. The individual is medically stable, willing, and capable of self‐management.
2. The treating clinician's familiarity with the insulin pump (including ward team), appropriate hospital policies/guidance on insulin pump use and inpatient diabetes management teams to support.

If a decision is made to continue with insulin pump therapy in hospital, this should be formally documented. A full list of prerequisites for safe use and contraindications of insulin pump can be found in Tables 5 and 6. Insulin pumps are expensive and any devices removed, should be labelled, stored in a safe place and documented.

**TABLE 5:** Requisites for safe perioperative use of subcutaneous insulin pumps (all conditions must be met).

Requisites for safe perioperative insulin pump use (all conditions must be met) The person with diabetes should be seen preoperatively by a registered health care practitioner who is knowledgeable about the perioperative use of insulin pumps Documentation of discussions and decisions made with the person with diabetes Multidisciplinary agreement that continued use of insulin pumps is appropriate Provision of an information leaflet for people in hospital Ability to communicate with medical teams Short fasting period (for example no more than one missed meal) Elective or expedited surgery Optimal preoperative HbA1c <69 mmol/mol where safe to achieve Ability to site pump away from the site of proposed surgery Ability to avoid positioning the insulin pump between the earthing plate and the diathermy Use of a Teflon® cannula (and not a steel cannula**)** Sufficient Teflon® consumables Ability to monitor CBG or via blood gas regularly (i.e. every 60 minutes) and to monitor capillary blood ketones Ability to replace subcutaneous insulin pump with variable rate intravenous insulin infusion (VRIII) if necessary

**TABLE 6:** Contraindications to insulin pump therapy in hospital. Other situations include where there is limited safety data to allow continued use of insulin pumps, for example during major surgery and as part of hybrid closed‐loop systems in hospital. Table adapted from Umpierrez et al.^17^ and Yeh et al.^36^

Contraindications to use of insulin pump in hospital Impaired level of consciousness or confusion Critical illness requiring intensive care/high dependency care Diabetic ketoacidosis or hyperosmolar hyperglycaemic state During MRI Psychiatric illness or suicidal ideation The individual is unable to use hands and/or physically manipulate pump due to medical condition The individual is unwilling to participate in diabetes self‐management, or share pump management decisions with hospital clinical staff Lack of pump supplies or mechanical pump malfunction Lack of trained healthcare providers or available diabetes specialists to supervise pump therapy Medical team decision for health and safety of the individual

Shortly after admission, inpatient diabetes teams and/or the outpatient diabetes service should be involved in helping with insulin adjustment and pump settings. Due to potential changes in an individual's condition during their hospital stay, the clinical team should reassess the person's ability to manage their diabetes device. If the individual is unable to do so, staff should assume responsibility and switch to alternative insulin delivery methods.

Errors in insulin pump management can potentially result in severe hypoglycaemia, hyperglycaemia or DKA that may not be caught by typical hospital safeguards, such as pharmacy review or scheduled point‐of‐care testing. Therefore, continued use of insulin pumps in the inpatient setting needs to be carefully considered. As ketosis may develop rapidly if insulin delivery from the pump is interrupted, it is essential that capillary ketone levels are checked if the CBG is elevated and corrective doses of rapidly acting insulin are given using an insulin pen. In the event of ketosis, the pump and delivery device should be checked for malfunction and the diabetes team immediately informed so that continued pump use be reconsidered and if necessary, a temporary intermittent subcutaneous insulin regimen be employed.

Specialist support is not usually available out of hours and may not be available at weekends–this lack of a 24/7 diabetes team and infrastructure to support in majority of UK hospitals remains a challenge to effective implementation within usual inpatient care. Thus, hospitals should introduce a hospital insulin pump policy, in addition to self‐management policies already in place,^30^ and should be provided with a detailed information sheet. An example used in the US has been published by Galindo et al.^31^

The concern with people on insulin pump therapy is they do not usually take any long‐acting insulin so if there is any interruption to insulin delivery (e.g. if the cannula is blocked or dislodged), hyperglycaemia and then ketoacidosis can develop very quickly. Errors in insulin pump management can potentially result in severe hypo‐ or hyperglycaemia that may not be caught by typical hospital safeguards, such as pharmacy review or scheduled point‐of‐care testing.

In these situations, the problem must be identified and rectified, for example by re‐siting the cannula, changing the tubing, or starting alternative insulin such as an intravenous infusion. Technical problems with the pumps can occur; the pump manufacturing companies offer round‐the‐clock telephone support and are typically able to provide a replacement pump within 24 h if required. All individuals using pumps are advised to retain a supply of their pre‐pump insulin pens for use in an emergency situation, for example, in case of pump failure or damage (Table 7).

**TABLE 7:** Starting basal insulin from insulin pump therapy (e.g. people who are unable to self‐manage their pump).

**Starting basal‐bolus insulin regimen**	
This is for people with T1D who are unable to self‐manage their insulin pump, but do not have unstable blood sugars, are not in a hyperglycaemic emergency and are not nil by mouth (NBM) A basal‐bolus regime is preferable to VRIII Nb: The insulin in an insulin pump is very short acting, and therefore, alternative insulin must be started immediately (i.e. within 1 h) to avoid risk of ketoacidosis Choice of basal insulin for the hospital admission should ideally include Lantus or Levemir. Ultra‐long acting insulins (Degludec or Glargine U‐300) may be more challenging when switching back to insulin pump therapy given their longer duration of action	
**Calculate Total Daily Dose (TDD)**	
**Method 1 Pump Total Daily Dose** (eg. 7 day average) Information can be obtained by individual or diabetes specialist nurse	**Method 2 Person's weight** Weight: kg × 0.5
**Basal and Bolus Insulin Dosing**	
**Basal Rate** (TDD × 0.5)	**Bolus (mealtime) insulin** (TDD × 0.5/3)
Prescribe 50% of the TDD as once daily Lantus (or can be divided in to twice daily Levemir)	For mealtime insulin (Novorapid/ Humalog/ Fiasp): 50% of TDD/3 plus a safety adjustment (e.g. minus 30%) to minimise risk of hypoglycaemia. Titrate doses according to response If the person is able to carbohydrate count, prescribe a variable rapid‐acting insulin dose for self‐adjustment
**Example:**	
A person's average pump insulin TDD for last 7 days is 48 units/day 50% of 48 units = 24 units as once daily Lantus insulin (or 12 units as twice daily Levemir insulin) 50% of 48 units/3 = 8 units of Novorapid with each meal: after safety adjustment = 6 units	

*Note*: Table adapted from Diabetes Technology Network (DTN) UK Best Practice Guidelines for managing continuous subcutaneous insulin infusion therapy in hospitalised patients.

Abbreviations: CSII, continuous subcutaneous insulin infusion; NBM; nil by mouth; TDD, total daily dose; T1D, type 1 diabetes; VRIII, variable rate intravenous insulin infusion.

#### 2.1.9 Practical recommendations: Insulin pump in the person able to self‐manage

Unless incapacitated, most people admitted to hospital using insulin pumps, who are physically and mentally able to continue to use their pumps, are safe to remain on insulin pump therapy. However, a significant proportion of medical staff will be unfamiliar with this technology. Specialist diabetes staff are required to be available to give advice and guidance if required.

If the individual is acutely unwell, unable or unwilling to manage the pump, unable to safely demonstrate pump settings, and/or no specialist advice is immediately available, the pump should be removed and a conventional intravenous insulin infusion or a subcutaneous basal‐bolus insulin regimen started.

It may not be possible for hospitals to have all the necessary insulin pump supplies, since each device may have different components that a hospital would not typically have on formulary. Therefore, the individual or family should be advised to provide the necessary pump supplies, such as infusion sets and insulin reservoirs. If supplies are not available, then insulin pump therapy will need to be discontinued whilst in hospital.

On admission, rapid‐acting insulin used in the pump reservoir should be prescribed for use in an insulin pump (either on the parenteral section of the drug chart or on the electronic record). For electronic insulin pump order sets, hospitals should alert the medical team on how to deal with pump discontinuation.

Individuals should be made aware of differing glucose targets in hospital, as it likely differs from outpatient goals.

Insulin pumps are expensive and steps should be taken to ensure they are not lost when a patient is admitted to hospital. The pump should only be adjusted by its owner (who has received extensive training) or a member of the diabetes team in possession of the correct knowledge and skills.

Table 4 summarises use of insulin pump use in special situations.

#### 2.1.10 Discontinuing/restarting insulin pumps

If the decision is made to discontinue the insulin pump on admission, individuals should be transitioned to a subcutaneous insulin injection regimen consisting of basal and bolus insulin, or continuous IV insulin infusion. Similarly, if an individual's medical condition changes during the hospital admission, and any concerns or a contraindication arises, insulin pump therapy should be discontinued. Examples include administered medications or analgesia, which may impair consciousness or cause confusion or development of renal injury or hepatic failure resulting in uraemia or encephalopathy.

Tables 7 and 8 summarise guidance for transferring individuals on CSII to/from subcutaneous insulin. When restarting insulin pump therapy after hospital admission with an acute illness, it is important to consider insulin needs during hospitalisation may greatly vary from pre‐admission levels and could also change after discharge.

**TABLE 8:** Restarting insulin pump therapy from basal IV/SC insulin. When restarting insulin pump therapy after hospital admission with acute illness, it is important to consider insulin needs during hospitalisation may greatly vary from pre‐admission levels and could also change after discharge.

**Insulin pump total daily dose (TDD) calculation**		
**Method 1 Pre‐Pump Total Daily Dose** Pre‐pump TDD × 0.75	**Method 2 Person's weight** Weight: kg × 0.5	
**Clinical considerations on pump TDD:** Average values from methods 1 and 2 Problematic hypoglycaemia: consider lower estimated TDD Hyperglycaemic, elevated HbA1c, or pregnant, consider higher estimated TDD		
**Pump Dose adjustment**		
**Basal Rate** (Pump TDD × 0.5)/24 h	**Carbohydrate Ratio (I:C) ratio** 400/TDD	**Insulin Sensitivity Factor (ISF)** 130/TDD
Start with one basal rate, adjust according to glucose values over basal rate testing Add additional basal according to need (e.g. Dawn phenomenon)	e.g. TDD 35 units = 400/35 = 11.4, I:C ratio 1 unit: 11 g Most adults require 1 unit: 8–15 g Acceptable post prandial rise is ~3 mmol/L Adjust based on low fat meals with known carbohydrate quantity	Correction insulin dose should bring glucose back to target range in 4–5 h
**Pump restart from basal SC insulin**		
Individual inserts new cannula performs a fixed prime and restarts insulin pump. The individual may need to temporarily reduce background insulin infusion rate (e.g. drop to a 70% temporary basal rate for 12‐24 h) while long‐acting subcutaneous insulin is still active, with increased glucose monitoring required (ideally use CGM). No further subcutaneous insulin doses should be required once insulin pump restarted. Capillary blood glucose should be checked 1–2 h after insulin pump restarted.		
**Pump restart from IV insulin**		
Individual inserts new cannula performs a fixed prime and restarts insulin pump (there is no need to wait until a meal) Wait 60 minutes before discontinuing IV insulin.		

*Note*: Table adapted from Diabetes Technology Network (DTN) UK Best Practice Guidelines for managing continuous subcutaneous insulin infusion therapy in hospitalised patients.

Abbreviations: ISF, insulin sensitivity factor; IV, intravenous; SC, subcutaneous; TDD, total daily dose.

#### 2.1.11 Hypoglycaemia whilst on an insulin pump

##### If able to manage their pump

Treat hypoglycaemia with 15 g rapid‐acting carbohydrates (e.g. dextrose tablets). Unlike people on long‐acting insulin, follow‐up with long‐acting carbohydrates is not usually needed. Pump infusion rates may need adjustment, especially if there is a history of recurrent hypoglycaemia: consult diabetes team.

##### If unconscious/incapacitated

Initial treatment of hypoglycaemia is as standard hospital policy. If persistent hypoglycaemia occurs, remove cannula and pump. Once normoglycaemic, restart insulin, either CSII if the person with diabetes is alert and able to self‐manage, or alternative regimen (see below); this is needed to prevent the development of ketoacidosis.

### 2.2 Hybrid closed‐loop systems/automated insulin delivery systems

Latest hybrid closed‐loop systems aim to minimise hypoglycaemia and hyperglycaemia and maintain glucose levels within a target range through use of a computerised algorithm to adjust the basal rate of insulin and administer corrective bolus doses. Unlike fully closed‐loop systems, the person with diabetes is still required to manually program insulin boluses with meals.

Healthcare professionals should be aware that not all systems with CGM and a pump are hybrid closed‐loop systems and these may work separately.

Insulin suspension via pump alone should not be used to treat hypoglycaemia because it does not work quickly enough.

#### 2.2.1 In‐hospital use of hybrid closed‐loop systems

There are limited data and guidance on real‐world use and safety of hybrid closed‐loop systems in the hospital setting. Within a research setting, improved percentage time in range, and lower mean glucose levels without increased hypoglycaemia^32^, ^33^ have been demonstrated in small, randomised trials with people with type 2 diabetes. There are also some case reports suggesting safe use in hospital.^34^, ^35^ Despite this evidence, the effect of hybrid closed‐loop systems on clinical outcomes, the best application of these devices, and cost‐effectiveness are yet to be determined.^36^ Furthermore, the safety profile for risk of hypoglycaemia/ketonaemia may be different. Finally, non‐diabetes specialists will be unfamiliar with these systems.

In someone who is well and may be in hospital for a short elective procedure or investigation, it may be appropriate to let the hybrid closed loop continue to control their glucose.

In someone who is unwell, insulin requirements can change rapidly from day to day and so JBDS‐IP recommend closed‐loop algorithms are ‘turned off’ and systems are switched to ‘manual’ mode in the hospital setting. This allows the individual with support from their diabetes team to adjust insulin pump settings, including glucose target range, insulin sensitivity factor, and basal rates.

Further challenging scenarios for hybrid close loop systems in hospital; include medications such as glucocorticoids, which may cause severe insulin resistance and uncontrolled hyperglycaemia, presenting a challenge for hybrid close loop algorithms. Other challenging scenarios include nausea and vomiting in people on hybrid close loop systems, and during periods of parental or enteral nutrition through nasogastric/percutaneous endoscopic gastrostomy (PEG) feed. In these scenarios, JBDS‐IP recommend these systems be changed to ‘manual mode’.

Individuals would need to meet the self‐management criteria as for normal continuation of their insulin pump in hospital. It is important to recognise that individuals may need adjustment in their basal rates when switched to standard insulin pump mode or basal insulin, and a review of glycaemia, pre‐admission basal rates, and number of automated insulin suspensions should be made. It is worth noting that some hybrid close loop systems, such as Omnipod 5®, may not provide specific details on basal patterns, but report the total amount of insulin given in the day. In such situations, conversion to multiple daily injections can be done through estimation from total daily insulin or weight calculations (Table 7).

For inpatients meeting the criteria to continue insulin pump and CGM therapy, continuing in closed‐loop mode is only recommended under specific guidance from the diabetes team.

After discontinuation of auto‐mode within the hybrid closed loop, the system may be used individually (as CGM only or insulin pump only). Continued use of these systems should be determined on a case‐by‐case basis, with shared decision‐making with individuals.

#### 2.2.2 Hybrid closed loop in surgery

Overall data on the safety or maximum safe duration of hybrid close loop control during anaesthesia is limited.^31^ Hybrid closed loops should only be used with the anaesthetist working in close collaboration with the diabetes inpatient teams, with close monitoring and a clear plan and knowledge of using these systems and what to do if glucose is out of range.

## 3 ELECTRONIC PRESCRIBING AND MEDICINES ADMINISTRATION

Electronic prescribing and medicines administration (EPMA) systems are designed to enhance the prescribing ordering and administrations of medicines and are in widespread use. The systems can improve inpatient care by supporting healthcare professionals to review and prescribe medications remotely from any location, improving flexibility and response times, as well as provide decision support and/or electronic prompts (Supplementary Material S2).

The challenges associated with EPR/EPMA systems include the complexity and time required to implement and learn. One of the major challenges is integration of glucose data (from CGM systems) into EPR. Additionally, the presence of hybrid EPR/EPMA systems, where prescriptions are electronic but patient data remains on paper, adds complexity and risk; a fully digital system will be safer and more efficient.

There is also a need to ensure system‐specific training, which can lead to delayed or incorrect prescriptions. Prescribing in dynamic situations (e.g. DKA/HHS/enteral feeding) can become cumbersome and require specific training. It is possible to mitigate some of this risk by building the necessary information into the local protocol for managing the condition.

Individuals that are self‐administering insulin at mealtimes may result in discrepancies on timings recorded on EPMA. Staff need to witness the administration and then record that it has been given. Care needs to be taken that the insulin is not recorded as given but the individual has not taken the medication.

The future areas of development for EPMA include enhancing interoperability with other health information systems (for example, CGM data). This would involve ensuring compatibility with a variety of healthcare databases and EHRs. There is also potential for significant advances with a more standardised approach to electronic prescribing, facilitating national data collection automatically, thereby streamlining care throughout the UK.

With the capability to monitor glucose through networked meters, EPMA systems are expected to integrate more tightly with individual metrics, triggering automatic reviews of medication in response to glucose fluctuations. Furthermore, embedding training within EPMA systems, particularly for high‐risk medications like insulin, could mandate or advise specific learning, thereby improving overall medication safety.

## 4 CLINICAL USE OF POINT‐OF‐CARE TESTING

Point‐of‐care testing (POCT) technologies are a key method of analysing blood, urine or other body fluid at the bedside or away from the routine laboratory. POCT measurements help facilitate decision‐making around diabetes management, but beyond this, have huge potential to drive improvements for inpatient safety, as well as enable rapid diagnosis or clinical decision‐making on the basis of results obtained in real time.

### 4.1 Point‐of‐care glucose measurements

Consideration should be given to the method of glucose analysis, when selecting a POCT glucose device, as there may be specific interferents. For example, compounds in dialysate fluid may interfere with some methods; a full description of assays and considerations for inpatient use has been reviewed.^37^

Ensuring that data from POCT devices is seamlessly integrated with Laboratory Information Management Systems (LIMS) and Electronic Health Records (EHR) through interfacing software (middleware) is crucial for maintaining data integrity, recording and linkage, and reducing clinical incidents. Without such connectivity, manual transcription of results can lead to increased risks of human error and jeopardise patient safety. Thus, total connectivity of the device to LIMS to EHR, or in some cases to LIMS and EHR independently, via middleware, is to be encouraged.

### 4.2 Using networked POCT measurements to drive clinical effectiveness

There are two key ways in which glucose measurements in the electronic health record can be used as follows:

1. Real‐time identification of people in hospital with extreme glucose, for example hyper‐ or hypoglycaemia, enables immediate intervention from specialised diabetes inpatient teams. Each hospital can set individualised glucose thresholds to suit each hospital trust.
2. Retrospective audit–In England, for example, hospitals are required to submit data on inpatient diabetes harms (i.e. National Diabetes Inpatient Safety Audit^38^). These data can be used to benchmark against audit standards but also reviewed serially to drive improvements as part of an audit cycle, in a given local service.

Clinical application of glucose and ketone POCT in UK hospitals varies significantly; while networked glucose meters are common, data is not always accessible by diabetes specialist teams, even when available. Time constraints may prevent effective use of the information, but the resource, infrastructure and staffing to embed these as a standard will be necessary.

## 5 INFORMATION TECHNOLOGY TO SUPPORT INPATIENT DIABETES CARE

Information technology in healthcare facilitates quicker access to clinical information, remote care, error monitoring, and service improvement through audit data. The use of systems that allow real time information enable diabetes teams to oversee a larger patient group. Furthermore, traditional telephone or paper‐based referrals can be replaced with instant electronic communication for faster responses.

Education and training of ward teams to improve diabetes care is an important role of the specialist team. Where undertaken electronically, this can be provided remotely, allowing more flexibility for the clinical staff, as well as a record of the diabetes team input to be available to the whole team at any time and the standard of care to be continuously monitored.

Use of these tools can potentially save the diabetes team a great deal of time. They will also increase the workload as we become aware of the need for specialist intervention for a much wider group of people with diabetes in hospital. Increased efficiency but also an increased workload may mean a larger diabetes inpatient team is needed to manage the work that has now been identified. The technology described above is currently available. The guidance on how the diabetes team can best use these tools is not. To make the most of these new tools requires different ways of working for the inpatient diabetes specialist team.

### 5.1 Electronic health records

An electronic health record (EHR; also known as electronic patient record or EPR) stores medical and nursing information and may also link to systems such as those storing radiology and laboratory results and patient administration systems. These systems may be developed in house by the hospital team or there are several commercial products available. Several of the commercial products were initially developed as hospital billing systems and may have the advantage of flagging specific conditions such as diabetes or recording diabetes emergencies such as diabetic ketoacidosis. There are specific advantages for the diabetes team in using an electronic health record. Most will combine the functions described above with the ability to record diabetes specific outcomes (for example, details of foot ulceration and a preventative care plan).

Current challenges of switching to an EHR include being challenging as the functionality may not exactly mirror the individual systems that they are replacing. Remote point‐of‐care glucose monitoring is unlikely to be part of the EHR package and may be challenging to incorporate directly. Careful thought needs to be given to ensure that care standards are maintained during the transfer period. The transfer to an EHR requires time to plan and implement, and diabetes teams are currently stretched by volumes of work and workforce shortages. This is hopefully a short‐term problem but there is the potential for larger teams to get better and smaller teams to get left further behind using outdated working practices. This can be addressed by developing national standards and ensuring that hospitals are appropriately supported to meet the standards.

## 6 SELF‐MANAGEMENT PROTOCOLS

Admission to hospital can disrupt glucose management for people with diabetes due to stress effect of illness, combined with changes in appetite, and changes in medications. This effect is magnified for people using technology like hybrid closed systems and sensors. Despite their expertise, self‐management can be taken away and overridden by staff with less experienced with using this technology. The National Institute of Clinical Excellence Quality Standards recommend that hospitals have and promote a self‐management policy, which supports people who want to self‐manage their T1D to safely do so while in hospital.

Consideration needs to be given to how this is linked to EPMA and EHR systems. For example, inpatient safety systems usually involve glucose readings from an organisation's own networked glucose meters, and readings not taken using these meters (such as from a CGM system or a person's own meter) will there bypass these systems. Until CGM data is EHR‐integrated, a suitable compromise may be for people to use both personal and hospital glucose monitoring systems concurrently.

### 6.1 Self‐management of insulin pumps (continuous subcutaneous insulin infusion during hospital admission)

Insulin pumps may be used by people with T1D to optimise glycaemia, with users undergoing detailed education and training by the diabetes specialist team.

### 6.2 Principles of self‐management of insulin pumps by inpatients

- Inpatients using insulin pumps should self‐manage if well enough to do so.
- If the person with diabetes is not well enough to self‐manage the pump or is unconscious/incapacitated, the pump should be discontinued and a variable rate intravenous insulin infusion should be commenced immediately.
- An insulin pump should NEVER be discontinued without immediate substitution of rapid‐acting insulin via an alternative administration route. The person using insulin pump therapy should already have an alternative basal/bolus insulin regimen in the event of pump failure.
- Insulin pumps should only be adjusted by the patient or a member of the diabetes team.
- If an insulin pump is discontinued it should be stored safely until the person with diabetes is ready to recommence the pump. The place of storage should be documented.
- If the person with diabetes is not able to self‐manage but continued intravenous insulin is not necessary, the diabetes specialist team should be asked to advise on a subcutaneous insulin injection regimen.
- The altered tissue perfusion in diabetic ketoacidosis (DKA) affects insulin absorption, making insulin pump therapy unreliable. The insulin pump should be temporarily discontinued in people with diabetes presenting in DKA: remove the cannula and detach the pump. For further management, follow standard DKA protocol.
- When an insulin pump is recommenced the intravenous insulin infusion should not be discontinued until a mealtime bolus dose of has been given via the pump.
- All people with diabetes admitted to hospital using an insulin pump should be referred to the diabetes specialist team.

### 6.3 Pump management for procedures requiring a period of starvation

- Continuous infusion of subcutaneous insulin via a pump is designed to maintain stable blood glucose during the fasting state.
- Procedures requiring the individual to be nil by mouth for a limited period (no more than one missed meal) may be manageable with a pump.
- Plans for continued use of the pump during an elective procedure should be discussed and agreed with the patient before admission.
- People with an insulin pump should not require overnight admission prior to the procedure.

### 6.4 Continuous glucose monitoring

The general principles described for insulin pump therapy also apply with CGM use. If the person with diabetes is capable and comfortable to continue using the device, then they should be allowed to do so. There is still a requirement to perform regular capillary glucose tests on a quality‐controlled hospital device to meet clinical governance standards.

If a person with diabetes attends hospital wearing one of these devices, then they should continue to use it if capable. At the current time routine capillary glucose and ketone testing will need to be performed in parallel by the ward team.

### 6.5 Electronic prescribing and medicines administration

Self‐administered medications need to be prescribed in a specific format to allow for variable doses of insulin being given and for insulin to be given at times that are different to the standard medication rounds. Both prescribers and those recording administration need to be specifically trained in this aspect of their hospital's electronic system. The inability of clinicians to use an electronic system must not be allowed to prevent an individual self‐administering medication. The inpatient diabetes specialist team should have the opportunity to input into developing appropriate processes for medication self‐administration, and may then be trained to provide specific support and training to other staff.

Systems need to be able to record the specific dose of insulin given at each injection. The timing that the dose was administered also needs to be recorded. This is especially important for short‐acting insulins that should be given at mealtimes, as these are unlikely to correspond with the timing of other medicines being administered. Ensuring that short‐acting insulins are given at mealtimes is particularly important for avoiding hypo‐ and hyper‐glycaemia.

Most systems will not allow people with diabetes to directly enter information. There is potential for adverse events if the information relating to timing and dose of insulin is not sought from the person with diabetes and entered in a timely way.

Hospital insulin safety groups need to specifically focus on electronic prescribing systems to ensure that they are contributing to improved insulin self‐administration and safety as focussed diabetes specialist input may be required particularly when systems are being introduced.

## 7 NATIONAL DATASETS TO IMPROVE DIABETES CARE

The National Diabetes Inpatient Audit (NaDIA) reviews several aspects of care including medication errors, inappropriate use of insulin infusions, harms such as in‐hospital hypoglycaemia, hospital acquired diabetic ketoacidosis and foot lesions.^38^ This paper‐based snapshot bedside audit involves the team visiting every inpatient with diabetes to examine their notes, prescription and observation charts. The first NaDIA report highlighted significant deficiencies in care, however, has subsequently shown several reductions in medication errors, inpatient hypoglycaemia and hospital acquired foot lesions. The process of national datasets and audits has raised the profile of inpatient diabetes care with other teams as well as hospital management, as well as supported business cases for investment into staff and weekend working.

NaDIA also collected data on use of technology in supporting inpatient diabetes care. Since its inception, there has been an increase in services using electronic patient records and electronic prescribing. The NaDIA has been an important lever in increasing the use of these devices, however, is currently paused in England and Wales. Despite its significant contribution to improving inpatient diabetes, it is recognised that NaDIA is labour intensive and costly.

Going forward, the National Diabetes Inpatient Safety Audit (NDISA) will be a valuable tool in extracting data from electronic patient records, electronic prescribing records, electronically collected harms such as hospital acquired diabetic foot lesions ulcers (from analysis of tissue viability pressure ulcer data) and frequency of severe hypoglycaemia from web‐linked glucose.^38^ The intention is to make this data available to hospitals on a monthly or quarterly dashboard for internal, as well as national comparison, to drive improvement in care.

In England, the Getting It Right First Time (GIRFT) programme is another important initiative, which has a focus on improving inpatient care for people with diabetes.^39^ The GIRFT diabetes programme involves visiting all hospitals in England to assess their diabetes services. This has resulted in a considerable levelling up of staffing and the use of technologies in inpatient care. Supporting this is another important initiative, the Diabetes Care Accreditation Programme (DCAP) launched by The Royal College of Physicians and Diabetes UK in May 2023.^40^ The programmed aims to improve inpatient care by setting quality standards and measuring how services perform against these, and is open to all hospitals in the UK. With respect to technology, use of electronic records, systems to identify all inpatients with diabetes and web‐linked glucose devices will be part of the assessment. Importantly self‐management of diabetes treatments and guidance on the use of wearable technologies during the inpatient stay will also form part of the assessment.

## 8 FUTURE RESEARCH

As technology for diabetes management continues to advance rapidly, further research is required within the hospital setting (Table 9). There are no current consensus guidelines on target CGM metrics in hospital, and further data (including long‐term outcomes) are required, including post myocardial infarction, stroke, or coronary artery bypass. Accuracy of continuous glucose monitoring is also required for specific hospital populations, particularly whilst people are acutely unwell in the emergency department (i.e. septic, febrile and/or hypotensive), during hyperglycaemic emergencies, in the intensive care setting and in people on haemodialysis or peritoneal dialysis. The role of CGM and hybrid closed‐loop systems in perioperative pathways also warrants further investigation.

**TABLE 9:** Key research areas for technology in hospital.

Key research areas Target CGM metrics in hospital Target CGM metrics and associated outcomes following myocardial infarction, stroke, coronary artery bypass Accuracy of CGM for specific hospital populations, that is acutely unwell in the emergency department (i.e. septic, febrile and/or hypotensive), during hyperglycaemic emergencies, in the intensive care setting Real‐world implementation on adjunctive and non‐adjunctive use of CGM and hybrid closed‐loop systems in hospital Clinical outcomes for CGM and hybrid closed‐loop systems on length of stay, in‐hospital complications and mortality Role of CGM and hybrid closed‐loop systems in perioperative pathway Role of CGM and hybrid closed‐loop systems in discharge from hospital pathway, including care homes and people at high risk Integration of continuous ketone monitoring combined with CGM Integration of CGM data within electronic health records and electronic prescribing systems with prompts Data standards and implementation policies to integrate CGM data into electronic health records. POC/CGM glucose linkage to national datasets Use of electronic health care records and POC data to identify people at high risk in hospital Health economics and costs of implementation of technology in hospital

Abbreviation: CGM, continuous glucose monitoring; POC, point of care.

Whilst evidence for hybrid closed‐loop systems for people in hospital is growing, clinical outcomes for different algorithms are yet to be determined particularly in people with type 1 diabetes. Integrated systems with continuous ketone monitoring could be combined with CGM technology to reduce rates of diabetic ketoacidosis, and aid in the management for people in hospital.^14^

CGM data are not currently integrated within the electronic health records of the user; therefore, there is a need for data standards and implementation policies to integrate CGM data into electronic health records. The iCoDE Project aims to facilitate standards towards this integration;^41^ however, it is imperative for hospitals, CGM manufacturers, and service providers for electronic health records to work together.^42^ Furthermore, the data acquisition process must be compliant with regulatory privacy rules and cybersecurity policies^42^ as users will need to provide consent their CGM data to be transferred to hospital health records. Future directions encompass integrating CGM data into electronic prescribing systems with prompts and enabling access for inpatient diabetes teams. Additional linkage to national datasets could facilitate hospital comparisons and drive improvement in care.

Finally, larger scale studies are required on whether CGM and hybrid closed‐loop systems can effectively improve in‐hospital glycaemia and reduce hospital length of stay, mitigate harms related to poor glycaemic control and enhance self‐management, and/or experience in hospital. Additionally, information is needed on costs of implementation of wearable diabetes technology for its widespread use in hospital. Increased costs may arise from repeated sensor malfunctions or the need for replacement in people undergoing MRI or other procedures. However, these costs are likely to be offset by the benefits of safer glycaemic control, reducing in hospital hypoglycaemia, hyperglycaemia and the potential resulting diabetic ketoacidosis or hyperosmolar hyperglycaemic syndrome.

## FUNDING INFORMATION

There is no funding to report.

## CONFLICT OF INTEREST STATEMENT

PA is in receipt of equipment from Dexcom for an investigator‐initiated research study. AL has received payments for speaking and advisory boards from Insulet, Dexcom, Abbott Diabetes Care, Novo Nordisk, Sanofi and Institutional Research Support from Abbott Diabetes Care, Novo Nordisk. DF is the national lead for the UK diabetes care accreditation programme and has received speaker honoraria from AstraZeneca, Novo Nordisk, and Sanofi Diabetes. SM is appointed to the Board of Trustees at the Diabetes Research & Wellness Foundation and is in receipt of funds from Dexcom for an investigator‐initiated research study. GR has received personal fees from Abbott Diabetes Care, Sanofi Aventis and Eli Lilly. PC has received personal fees from Abbott Diabetes Care, Dexcom, Ypsomed, Vertex, Glooko, Eli Lilly, Insulet, Medtronic, Novo Nordisk, Roche and Sanofi Aventis. KD is the immediate past chair of the Joint British Diabetes Societies for Inpatient Care and has received speaker fees, travel or taken part in advisory boards for AstraZeneca, Sanofi Diabetes, Boehringer Ingelheim, Lilly, Menarini, and Novo Nordisk.

## ADDITIONAL WEBSITE LINKS

JBDS‐IP guidelines: https://abcd.care/jbds‐ip.

DTN guidance: https://abcd.care/dtn/best‐practice‐guides.

Centre for Perioperative Care (CPOC). Perioperative Care of People with Diabetes Undergoing Surgery. https://www.cpoc.org.uk/guidelines‐resources‐guidelines‐resources/guideline‐diabetes.

Diabetes Accreditation Care Programme‐Royal College of Physicians https://www.dcap.org.uk/.

Association of British Clinical Diabetologists (ABCD)‐Position Papers and Guidelines https://abcd.care/position‐papers‐and‐guidelines.

## Supporting information


Appendix S1:

## Data Availability

Data sharing not applicable to this article as no datasets were generated or analysed during the current study.
