# Supplementary material for: Using technology to support diabetes care in hospital: Guidelines from the Joint British Diabetes Societies for Inpatient Care (JBDS‐IP) group and Diabetes Technology Network (DTN) UK
Source: Diabet Med. 2024 Oct 21;42(3):e15452. doi: 10.1111/dme.15452 (PMC11823341; doi:10.1111/dme.15452)
Supplement: Supplementary file 1 — Appendix S1: [file DME-42-e15452-s001.docx]

# Supplementary Material 1: Reviewing CGM data

Some people will automatically be sharing glucose data from their CGM sensor with the diabetes team, which enables the diabetes team to remotely monitor the individual’s glucose levels. On some devices, it is possible to review glucose data using the “history” function on the device to review glucose levels over the last 7-90 days. This allows the clinician to understand the person’s usual glycaemia. However, as these systems require sensors to be linked, this may not always be possible in the acute setting.

Importantly, automated uploading of data is only available for people who have registered for these platforms; and is not available for those who use a reader (who can upload data intermittently by plugging the reader into an appropriate internet connected device).

Examples of platforms to review CGM data include, but are not limited to:

- Dexcom data linked to Dexcom Clarity (<https://clarity.dexcom.eu/professional/>)

- Freestyle Libre data linked to Libre view (<https://www.libreview.com/>)

- Medtronic pump or CGM data linked to Carelink (<https://carelink.minimed.com/>)

Other generic platforms include:

- Glooko (https://glooko.com/)

Currently the technology yet needs to evolve for data to be available from multiple patients from a single device.

# Supplementary Material 2: Benefits of EPMA systems

| For pharmacy | - Reduction in prescribing errors through use of mandatory fields such as allergies and sensitivities and decision support (for example ensuring that insulin is prescribed in units) - Saving in time as a medication chart does not need to be transported to pharmacy or the pharmacist visit the ward to view the chart - Legible prescribing saving time and preventing errors - A clear record and auditable trail of what medication has been given and when |
| --- | --- |
| For the ward team | - Provides a legible prescription that is available to the whole clinical team - Ward team can be supported at a distance by other specialists such as pharmacy - Regular ward level audits of diabetes medication prescription and administration can be established - Other examples of decision support include: - Links to protocols for use on VRIII, DKA and HHS when intravenous insulin is being prescribed. - Alert sent to the diabetes team when intravenous insulin is prescribed. - Links to e-learning module on safe use of insulin for any insulin prescription - Limiting the prescription of short acting insulin to mealtimes and correction doses - Alerts if medication needs reviewing |
| For the diabetes team | - Records and medication can be viewed at a distance from the ward, enabling diabetes specialist time to be focussed on those individuals who will benefit most - Enables certain groups of people with diabetes to be identified (e.g. if admitted with diabetic ketoacidosis) - Matching individual glucose levels to the dosing and timing of diabetes medication should be easier as the specific time that the medication is administered is recorded. - As real time audits of ward level data on prescription and medication errors are available it is possible to plan specific training for areas that require it. |
| For the person on the ward with diabetes | - Safer care - A more responsive service - Reducing prescription errors - Potential improvement in the speed of the discharge process |

# Supplementary Material 3: Audit standards

The technology used in this document is primarily there to assist in providing good care in hospital. It is therefore part of a process rather than an outcome. The technology described can greatly assist in measuring quality of care rather than being something that is measured itself. For example, EPMA systems can produce automated monthly reports of prescribing or administration errors relating to insulin use. Electronic health records can produce automated reports that then feed into national audits comparing quality of care (an example being the national audit of diabetic ketoacidosis). There are also some specific audits of safety aspects of technology that should be measured. The list below is illustrative but not exhaustive.

|  | **Audit standard** |
| --- | --- |
| **Safe use of wearable technology on admission to hospital:**   - Percentage of ward staff that can recognize an insulin pump or glucose sensor - Percentage of staff that can access guidelines for the use of wearable technology - Percentage of staff that are aware of what to do if an individual is admitted acutely unwell using wearable diabetes technology.   Audit of clinical incidents relating to safe use of wearable technology in hospital | 100%  100%  100% |
| **Audit of staff training in the prescribing and administration of insulin:**   - Percentage of relevant staff that have completed mandatory training. - Percentage of staff that successfully completed a test of safe prescribing | 100%  80%  70% |
| All hospitals should be completing patient satisfaction questionnaires at regular intervals. Ability to use their wearable technology should be part of this audit | N/A |
| Organisations should have a diabetes database that supports electronic data identification (and submission where appropriate) for national diabetes audits | **-** |
